# *FCGR2A* and *FCGR3A* polymorphisms and clinical outcome in metastatic colorectal cancer patients treated with first-line 5-fluorouracil/folinic acid and oxaliplatin +/- cetuximab

**DOI:** 10.1186/1471-2407-14-340

**Published:** 2014-05-19

**Authors:** Janne B Kjersem, Eva Skovlund, Tone Ikdahl, Tormod Guren, Christian Kersten, Astrid M Dalsgaard, Mette K Yilmaz, Tone Fokstuen, Kjell M Tveit, Elin H Kure

**Affiliations:** 1 Department of Genetics, Institute for Cancer Research, Oslo University Hospital, Postboks 4953 Nydalen, 0424 Oslo, Norway; 2 School of Pharmacy, University of Oslo and the Norwegian Institute of Public Health, Oslo, Norway; 3 Department of Oncology, Oslo University Hospital, Oslo, Norway; 4 Center for Cancer Treatment, Southern Hospital Trust, Kristiansand, Norway; 5 Department of Oncology, Aalborg University Hospital, Aalborg, Denmark; 6 Department of Oncology, Karolinska University Hospital, Stockholm, Sweden

**Keywords:** Colorectal cancer, *FCGR2A*, *FCGR3A*, Polymorphism, Cetuximab

## Abstract

**Background:**

Polymorphisms of genes encoding the Fcy receptors (Fc fragment of IgG receptor 2A (*FCGR2A*) and 3A (*FCGR3A*)), which influence their affinity for the Fc fragment, have been linked to the pharmacodynamics of monoclonal antibodies. Most studies have been limited by small samples sizes and have reported inconsistent associations between the *FCGR2A* and the *FCGR3A* polymorphisms and clinical outcome in metastatic colorectal cancer (mCRC) patients treated with cetuximab. We investigated the association of these polymorphisms and clinical outcome in a large cohort of mCRC patients treated with first-line 5-fluorouracil/folinic acid and oxaliplatin (Nordic FLOX) +/- cetuximab in the NORDIC-VII study (NCT00145314).

**Methods:**

504 and 497 mCRC patients were evaluable for the *FCGR2A* and *FCGR3A* genotyping, respectively. Genotyping was performed on TaqMan ABI HT 7900 (Applied Biosystems, Foster City, CA, USA) with pre-designed SNP genotyping assays for *FCGR2A* (rs1801274) and *FCGR3A* (rs396991).

**Results:**

The response rate for patients with the *FCGR2A* R/R genotype was significantly increased when cetuximab was added to Nordic FLOX (31% versus 53%, interaction *P* = 0.03), but was not significantly different compared to the response rate of patients with the *FCGR2A* H/H or H/R genotypes given the same treatment. A larger increase in response rate with the addition of cetuximab to Nordic FLOX in patients with *KRAS* mutated tumors and the *FCGR2A R/R* genotype was observed (19% versus 50%, interaction *P* = 0.04). None of the *FCGR3A* polymorphisms were associated with altered response when cetuximab was added to Nordic FLOX (interaction *P* = 0.63). Neither of the FCGR polymorphisms showed any significant associations with progression-free survival or overall survival.

**Conclusion:**

Patients with *KRAS* mutated tumors and the *FCGR2A* R/R polymorphism responded poorly when treated with chemotherapy only, and experienced the most benefit of the addition of cetuximab in terms of response rate.

## Background

The prognosis for patients with metastatic colorectal cancer (mCRC) remains poor even though the addition of newer chemotherapeutic agents and targeted drugs has increased the median survival from 12 months with fluorouracil monotherapy to roughly 2 years [1]. Cetuximab, a monoclonal antibody targeting the epidermal growth factor receptor (EGFR), has shown efficacy in combination with chemotherapy or given as monotherapy in a small fraction of mCRC patients [2]. Clinical benefit seems to be restricted to patients with *KRAS* wild-type tumors [3,4]. In the recent NORDIC-VII study, however, we did not find an improved outcome of adding cetuximab to first-line oxaliplatin-based chemotherapy in patients with *KRAS* wild-type tumors [5]. Similar results were found by the COIN trial and the recent EPOC study [6,7]. The results of these trials demonstrate the necessity to explore predictive markers independent of *KRAS* status to avoid unnecessary drug toxicity and reduce treatment cost.

Cetuximab may exert its antitumor effect through multiple mechanisms. One mechanism of its antitumor effects is through antibody-dependent cellular cytotoxicity (ADCC) [8]. ADCC is induced through the interaction of the Fc region of the monoclonal antibody with the Fc gamma receptor (FCGR), surface receptors for immunoglobulin G (IgG), located on immune effector cells such as natural killer lymphocytes and macrophages [9]. Polymorphisms have been demonstrated on genes encoding for the receptors *FCGR2A* and *FCGR3A*, affecting their affinity to human IgG: a histidine(H)/arginine(R) polymorphism at position 131 for *FCGR2A* and a valine (V)/phenylalanine (F) polymorphism at position 158 for *FCGR3A*[10]. The polymorphisms have been reported to be associated with clinical outcome to the monoclonal antibodies rituximab [11,12] and trastuzumab [13,14] in the treatment of lymphoma and breast cancer, respectively.

Previous studies exploring these polymorphisms in relation to cetuximab effect in mCRC have shown conflicting results and have been dominated by low-powered studies. The aim of the present study was to investigate the association between these polymorphisms and the effect of cetuximab treatment in a large mCRC patient cohort; the NORDIC-VII cohort. We examined the *FCGR2A* and *FCGR3A* polymorphisms as potential markers to predict cetuximab effect in 504 and 497 evaluable mCRC patients, respectively, treated with conventional chemotherapy (Nordic FLOX) with and without the addition of cetuximab.

## Methods

### NORDIC VII

In the NORDIC VII trial (NCT00145314, registered September 2, 2005), a total of 571 patients with mCRC were randomized to receive first-line standard Nordic FLOX (bolus 5-fluorouracil/folinic acid and oxaliplatin) (arm A), cetuximab and Nordic FLOX (arm B), or cetuximab combined with intermittent Nordic FLOX (arm C). Primary endpoint was progression-free survival (PFS). Overall survival (OS) and response rate were secondary endpoints. DNA from primary tumors was screened for the presence of seven *KRAS* mutations (codons 12 (G12D, G12A, G12V, G12S, G12C, G12R) and 13 (G13D)) and one *BRAF* (*BRAF* V600E) mutation as previously described [5]. *KRAS* and *BRAF* mutation analyses were obtained in 498 (88%) and 457 patients (81%), respectively. *KRAS* mutations in codons 12 and 13 were found in 39% of the tumors. *BRAF* mutations (V600E) were present in 12% of the tumors. The mutational frequencies of the 195 *KRAS* mutations in the NORDIC VII cohort were; G12A (9.7%), G12R (1.5%), G12D (35.4%), G12C (9.7%), G12S (6.2%), G12V (15.4%), and G13D (22.1%). Cetuximab did not add significant benefit to Nordic FLOX and *KRAS* mutation was not predictive for cetuximab effect. DNA from a total of 504 and 497 of the 566 patients in the intention to treat population was evaluable for the *FCGR2A* and *FCGR3A* genotyping, respectively. There were 172 patients in arm A and 332 patients in arms B and C evaluable for response and survival analyses for the *FCGR2A* polymorphism. There were 169 patients in arm A and 328 patients in arms B and C evaluable for response and survival analyses for the *FCGR3A* polymorphism. *KRAS* status was available from 442 and 437 patients with *FCGR2A* and *FCGR3A* status, respectively. *BRAF* status was available from 410 and 405 patients with *FCGR2A* and *FCGR3A* status, respectively. Response status was evaluated according to the RECIST version 1.0 criteria and was assigned to patients with complete or partial remission with changes in tumor measurements confirmed by repeat studies performed no less than 4 weeks after the criteria for response were first met (minimal interval of 8 weeks – 4 cycles) [15]. The study was approved by national ethics committees and governmental authorities in each country and was conducted in accordance with the Declaration of Helsinki. All patients provided written informed consent.

Primary tumors in the NORDIC VII study were screened for *KRAS* exon 2 (codons 12 and 13) mutations. However, recent studies have demonstrated that wild-type *RAS* should be defined by the absence of *KRAS* exons 2, 3, and 4 mutations and the absence of *NRAS* exons 2, 3, and 4 mutations [16-18]. A follow-up study of the NORDIC VII cohort will include these additional mutational analyses.

### FCGR2A-H131R and FCGR3A-V158F genotyping

Genotyping was performed on a TaqMan ABI HT 7900 (Applied Biosystems, Foster City, CA, USA) with pre-designed SNP genotyping assays for FCGR2A c.535A > G (rs1801274; resulting in amino-acid change of histidine to arginine at position 131) and FCGR3A c.818A > C (rs396991; resulting in amino-acid change of valine to phenylalanine at position 158), according to the manufacturer’s protocol. Negative controls (water) were included.

### Statistical analyses

The *χ*^2^-test and one-way ANOVA were used to compare categorical and continuous variables between groups, as appropriate, respectively. Homoscedasticity was ascertained and the non-parametric Kruskal-Wallis test was applied as a sensitivity analysis. For the prognostic analyses all three arms (arms A, B and C) were analyzed together. For the predictive analyses of cetuximab effect by *FCGR2A* or *FCGR3A* genotype, arm A was compared to arms B and C combined. The associations between the *FCGR2A* and *FCGR3A* genotypes and tumor response were analyzed by binary logistic regression. PFS and OS times were estimated using the Kaplan-Meier method. The associations of the *FCGR2A* and *FCGR3A* genotypes and PFS and OS were analyzed by Cox’s proportional hazards model. The assumption of proportional hazards was checked by inspection of log minus log plots. The potential value of *FCGR2A* and *FCGR3A* as predictive markers of cetuximab effect was analyzed by including an interaction term in the models. The distributions of the *FCGR2A* and *FCGR3A* genotypes in the NORDIC-VII study were tested for Hardy-Weinberg equilibrium [19]. *P* < 0.05 was considered statistically significant. All statistical analyses were performed using Statistical Package for Social Sciences, version 18.0 (SPSS Chicago, IL).

## Results

### Patient characteristics

Table 1 depicts the frequencies of the analyzed *FCGR2A* and *FCGR3A* genotypes, which were in Hardy-Weinberg equilibrium (*P* = 0.41 and 0.54, respectively). There were no significant associations of any of the *FCGR2A* or *FCGR3A* genotypes with clinicopathological characteristics (age, sex, location of primary tumor, metastatic sites, *KRAS,* or *BRAF* mutation status) or treatment, Table 2.

**Table 1:** ***FCGR2A *** **and *FCGR3A *** **genotypes in the study population**

	**Actual frequency of genotypes (n)**	**Expected frequency of genotypes (n)**	**Hardy-Weinberg equilibrium *X***^**2 **^**(1 degree of freedom)**	***P*-value**
***FCGR2A***				
H/H	114	118.61	0.68	0.41
H/R	261	251.78		
R/R	129	133.61		
***FCGR3A***				
F/F	241	238.10	0.37	0.54
F/V	206	211.8		
V/V	50	47.10		

**Table 2:** **Patient characteristics and treatment outcome by *FCGR2A *** **and *FCGR3A *** **genotypes**

	***FCGR2A***				***FCGR3A***			
	***H/H***	***H/R***	***R/R***	***P*-value**	***F/F***	***F/V***	***V/V***	***P*-value**
Number of patients (%)	114 (22.6%)	261 (51.8%)	129 (25.6%)		241 (48.5%)	206 (41.4%)	50 (10.1%)	
Age, median (range)	61 (27–74)	62 (24–75)	62 (30–75)	0.99^*^	62 (24–75)	61 (29–75)	61 (35–75)	0.47^*^
Sex, female/male	49/65	102/159	57/72	0.58^†^	93/148	94/112	20/30	0.32^†^
Location, colon/rectum	71/43	145/116	78/51	0.41^†^	147/94	121/85	23/27	0.15^†^
Metastatic sites, 1/>1	32/82	63/198	39/90	0.41^†^	62/179	51/155	20/30	0.08^†^
*KRAS*, wt/mutated	62/38	150/82	61/49	0.26^†^	126/87	111/67	31/15	0.54^†^
*BRAF*, wt/mutated	80/11	192/26	91/10	0.88^†^	182/18	141/21	36/7	0.28^†^
Treatment, FLOX/FLOX + cetuximab	33/81	90/171	49/80	0.34^†^	79/162	75/131	15/35	0.58^†^
Response; response/no-response	54/60	121/140	57/72	0.89^†^	109/132	93/113	25/25	0.82^†^
PFS (months), median	8.3	7.9	7.6	0.45^‡^	7.9	7.6	8.4	0.76^‡^
OS (months), median	21.9	19.8	18.2	0.42^‡^	19.9	20.5	19.7	0.77^‡^

^*^One-way ANOVA (The Kruskal-Wallis test produced similar p-values),^†^Chi-square test ^‡^Log-rank test.

### Response rate and survival

There was no significant difference in response rates for the different *FCGR2A* and *FCGR3A* genotypes when analyzing all the three treatment arms together (*P* = 0.89 and 0.82, respectively), Table 2. There was also no significant association of any of the *FCGR2A* or *FCGR3A* genotypes with PFS (Log rank *P* = 0.45 and 0.76, respectively) or OS (Log rank *P* = 0.42 and 0.77, respectively), Table 2.

### Predictive analyses for benefit of cetuximab treatment

**Figure 1:**
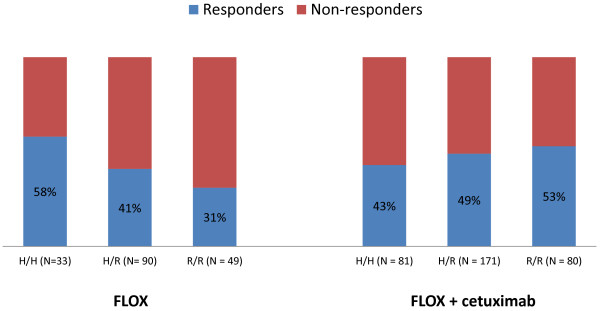
***FCGR2A *response rates in the whole study population.** The *FCGR2A* R/R genotype was associated with increased response rate when cetuximab was added to Nordic FLOX (31% in arm A vs 53% in arms B + C, interaction *P* = 0.03).

**Figure 2:**
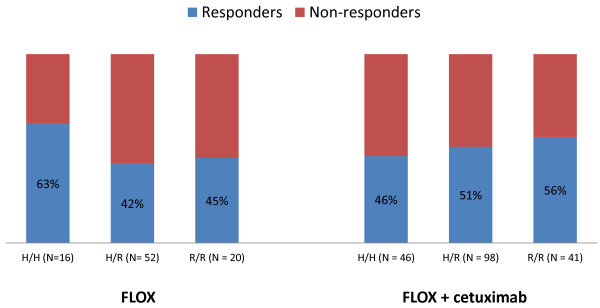
***FCGR2A *response rates in patients with *KRAS *wild-type tumors.** There was no significant difference in response rates when cetuximab was added to Nordic FLOX in the different *FCGR2A* subgroups (interaction *P* = 0.27).

**Figure 3:**
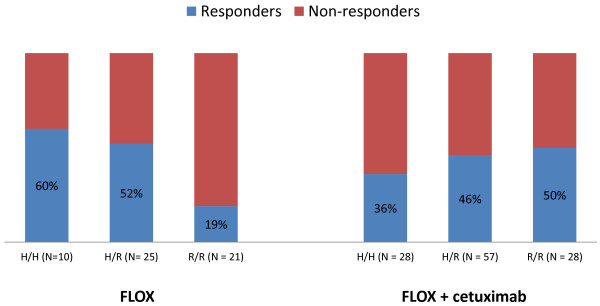
***FCGR2A *response rates in patients with *KRAS *mutated tumors.** The *FCGR2A* R/R genotype was associated with increased response rate when cetuximab was added to Nordic FLOX (19% in arm A vs 50% in arms B + C, interaction *P* = 0.04).

The *FCGR2A* R/R genotype was associated with increased response rate when cetuximab was added to Nordic FLOX regardless of mutational status (31% in arm A versus 53% in arms B + C, interaction *P* = 0.03), but was not significantly different compared to the response rate of patients with the *FCGR2A* H/H or H/R genotypes given the same treatment, Table 3 and Figure 1. There was no significant difference in response rates in the *FCGR2A* subgroups in patients with *KRAS* wild-type tumors after the addition of cetuximab, Table 4 and Figure 2. A significant increase in response rate with the addition of cetuximab to Nordic FLOX in patients with *KRAS* mutated tumors and the *FCGR2A R/R* genotype was observed (19% versus 50%, interaction *P* = 0.04), Table 4 and Figure 3. None of the *FCGR3A* polymorphisms were associated with altered response when cetuximab was added to Nordic FLOX (interaction *P* = 0.63), Table 3. The *FCGR3A* genotypes were not associated with response to cetuximab when stratified for *BRAF* or *KRAS* mutational status, Table 5.

**Table 3:** **Treatment outcome by *FCGR2A *** **and *FCGR3A *** **genotypes, and therapy received**

	**FLOX**			**FLOX + cetuximab**			**Interaction *P*-value**
***FCGR2A***	***H/H***	***H/R***	***R/R***	***H/H***	***H/R***	***R/R***	
Number of patients	N = 33	N = 90	N = 49	N = 81	N = 171	N = 80	
Response (%)	58% (19/33)	41% (37/90)	31% (15/49)	43% (35/81)	49% (84/171)	53% (42/80)	0.03^*^
PFS, median (months)	8.4	7.9	7.5	8.3	7.8	7.6	0.35^†^
OS, median (months)	28.0	20.5	19.8	21.4	19.5	17.3	0.85^†^
***FCGR3A***	***F/F***	***F/V***	***V/V***	***F/F***	***F/V***	***V/V***	
Number of patients	N = 79	N = 75	N = 15	N = 162	N = 131	N = 35	
Response (%)	38% (30/79)	41% (31/75)	53% (8/15)	49% (79/162)	47% (62/131)	49% (17/35)	0.63^*^
PFS, median (months)	7.6	8.4	7.8	8.1	7.4	9.3	0.41^†^
OS, median (months)	20.4	20.5	19.7	19.7	21.1	20.1	0.78^†^

^*^Logistic regression, ^†^Cox proportional hazard model.

**Table 4:** **Treatment outcome by *FCGR2A *** **genotype, *KRAS *** **or *BRAF *** **mutational status, and therapy received**

	**FLOX**			**FLOX + cetuximab**			
	***H/H***	***H/R***	***R/R***	***H/H***	***H/R***	***R/R***	**Interaction *P*-value**
***KRAS *** **wild-type (N = 273)**							
Number of patients	N = 16	N = 52	N = 20	N = 46	N = 98	N = 41	
Response	63% (10/16)	42% (22/52)	45% (9/20)	46% (21/46)	51% (50/98)	56% (23/41)	0.27^*^
PFS	8.4	8.9	9.0	7.7	7.7	8.0	0.23^†^
OS	31.6	23.6	19.0	21.4	20.7	18.9	0.23^†^
***KRAS *** **mutated (N = 169)**							
Number of patients	N = 10	N = 25	N = 21	N = 28	N = 57	N = 28	
Response	60% (6/10)	52% (13/25)	19% (4/21)	36% (10/28)	46% (26/57)	50% (14/28)	0.04^*^
PFS	8.1	8.3	7.1	7.7	8.1	6.7	0.90^†^
OS	17.2	20.4	24.3	21.1	20.0	16.8	0.34^†^
***BRAF *** **wild-type (N = 363)**							
Number of patients	N = 22	N = 62	N = 34	N = 58	N = 130	N = 57	
Response	64% (14/22)	48% (30/62)	35% (12/34)	47% (27/58)	52% (68/130)	54% (31/57)	0.10^*^
PFS	9.3	8.9	7.7	8.5	8.1	8.0	0.47^†^
OS	31.6	23.8	21.5	21.9	21.5	17.6	0.93^†^
***BRAF *** **mutated (N = 47)**							
Number of patients	N =3	N = 10	N = 4	N = 8	N = 16	N = 6	
Response	33% (1/3)	20% (2/10)	0% (0/4)	13% (1/8)	25% (4/16)	33% (2/6)	0.72^*^
PFS	4.3	5.1	3.8	3.8	4.6	5.8	0.36^†^
OS	9.2	9.4	5.6	8.9	8.1	11.3	0.73^†^

^*^Logistic regression, ^†^Cox proportional hazard model.

**Table 5:** **Treatment outcome by *FCGR3A *** **genotype, *KRAS *** **or *BRAF *** **mutational status, and therapy received**

	**FLOX**			**FLOX + cetuximab**			
	***F/F***	***F/V***	***V/V***	***F/F***	***F/V***	***V/V***	**Interaction *P*-value**
***KRAS *** **wild-type (N =268)**							
Number of patients	N = 39	N = 38	N = 9	N = 87	N = 73	N = 22	
Response	44% (17/39)	45% (17/38)	56% (5/9)	48% (42/87)	51% (37/73)	55% (12/22)	0.95^*^
PFS	7.8	9.0	8.4	8.0	7.3	11.8	0.72^†^
OS	23.1	20.5	25.2	17.6	25.9	20.5	0.97^†^
***KRAS *** **mutated (N =169)**							
Number of patients	N = 28	N = 21	N = 6	N = 59	N = 46	N = 9	
Response	36% (10/28)	48% (10/21)	50% (3/6)	51% (30/59)	39% (18/46)	33% (3/9)	0.28^*^
PFS	7.8	8.1	4.0	8.3	7.0	6.9	0.19^†^
OS	18.5	24.3	17.1	21.3	17.7	16.4	0.63^†^
***BRAF *** **wild-type (N = 359)**							
Number of patients	N = 56	N = 47	N = 12	N = 126	N = 94	N = 24	
Response	45% (25/56)	49% (23/47)	50% (6/12)	52% (65/126)	48% (45/94)	63% (15/24)	0.71^*^
PFS	7.9	9.1	7.8	8.3	7.6	11.5	0.58^†^
OS	23.8	23.6	19.7	20.6	22.9	20.5	0.93^†^
***BRAF *** **mutated (N = 46)**							
Number of patients	N = 8	N = 7	N = 2	N = 10	N = 14	N = 5	
Response	13% (1/8)	14% (1/7)	50% (1/2)	20% (2/10)	29% (4/14)	0% (0/5)	0.99^*^
PFS	5.9	4.3	4.4	4.2	5.4	4.6	0.87^†^
OS	9.5	9.4	5.2	10.8	8.9	10.3	0.66^†^

^*^Logistic regression, ^†^Cox proportional hazard model.

Median PFS and OS were similar in arms B + C as compared to arm A for the *FCGR2A* (Log rank *P* = 0.35 and 0.85) and the *FCGR3A* (Log rank *P* = 0.41 and 0.78) genotypes, Table 3. The median PFS and OS were also similar in arms B + C compared to arm A for both the *FCGR2A* and *FCGR3A* genotypes when stratified for *BRAF* or *KRAS* mutational status, Tables 4 and 5.

## Discussion

We studied the *FCGR2A* and the *FCGR3A* polymorphisms in a large cohort of mCRC patients treated with conventional chemotherapy with and without cetuximab in an effort to explore potential associations between these polymorphisms and cetuximab effect. Our results show that the addition of cetuximab to Nordic FLOX lead to a statistically significant increase in response rate in patients with the *FCGR2A* R/R genotype. Subgroup analysis of patients with *KRAS* mutated tumors and the *FCGR2A* R/R genotype showed an even larger increase in response after the addition of cetuximab.

Previous studies exploring the relation between the FCGR polymorphisms and cetuximab efficacy in mCRC have demonstrated conflicting or negative results and have been mostly low-powered studies with small sample sizes. Our study is one of the largest reported so far and unlike most of the other studies we included a control group where patients did not receive cetuximab.

Even though their results were not statistically significant, the *FCGR2A* R/R genotype had a better response rate compared to the H/R or the H/H genotypes in *KRAS* wild-type patients treated with cetuximab or panitumumab as monotherapy or in combination with chemotherapy in a study of 104 refractory mCRC patients [20]. Furthermore, a pooled analysis including 217 mCRC patients treated with cetuximab alone or with chemotherapy showed that patients with the *FCGR2A* R/R or H/R alleles had a statistically significant longer median PFS than the H/H genotype [21]. Moreover, a study by Negri et al*.*, where most of the 86 mCRC patients enrolled in the study were treated with cetuximab and irinotecan, demonstrated a higher OS in mCRC patients with the *FCGR2A* R/R polymorphism [22]. However, the authors concluded that the polymorphism was not predictive of cetuximab effect since no relation to response or time to progression (TTP) was demonstrated [22].

Conversely, a study which included 69 mCRC patients reported the *FCGR2A* H/H alone or in combination with *FCGR3A* V/V to be associated with longer PFS in irinotecan-refractory mCRC patients with *KRAS* wild-type and *KRAS* mutated tumors treated with cetuximab plus irinotecan [23]. The difference remained significant for *KRAS* mutated patients. Similar results were demonstrated by Rodriguez et al., who reported that patients with any *FCGR2A* H and/or *FCGR3A* V allele were more likely to show a response or have stable disease [24]. Rodriguez et al. explored if the FCGR genotypes would predict which patients with a *KRAS*, or other downstream mutations, would respond to cetuximab. They included 47 mCRC patients treated with cetuximab and standard chemotherapy with a *KRAS*, *BRAF*, *NRAS*, or *PI3K* mutation in the FCGR genotype analysis. Two other studies including 52 and 49 mCRC patients, respectively, reported only the *FCGR3A* V/V genotype to be associated with a better response to cetuximab [25,26].

In contrast, three other studies including 65, 58, and 122 mCRC patients, respectively, have reported the *FCGR3A* F/F allele to be associated with a better clinical outcome [27-29]. The former study demonstrated that patients enrolled in the BOND-2 study with the *FCGR3A* F/F allele had a significantly better response to cetuximab in combination with bevacizumab in irinotecan-refractory mCRC patients [27]. There was shorter survival in patients with the *FCGR3A* V/V genotype as compared to V/F or F/F in the study of 58 mCRC patients who received irinotecan in combination with cetuximab [28]. This was shown in the whole study population and in a subgroup analysis of patients with *KRAS* wild-type tumors. Moreover, the latter study by Pander et al., found mCRC patients in the CAIRO2 study with the *FCGR3A* F/F allele to be associated with longer PFS in *KRAS* wild-type patients treated with cetuximab as first-line treatment in combination with capecitabine, oxaliplatin and bevacizumab [29]. A smaller study including only 39 mCRC patients reported the *FCGR2A*, any H allele, and *FCGR3A*, any F allele, to be associated with longer PFS in mCRC patients who were treated with single-agent cetuximab [30]. These results could though not be replicated when the sample size was increased to a total of 130 patients [31]. In addition to the study by Lurje et al., four other studies with a higher number of patients have reported lack of significant associations of the *FCGR2A* or *FCGR3A* polymorphisms and cetuximab efficacy in mCRC [20,32-34].

Our study show that patients with *KRAS* mutated tumors and the *FCGR2A* R/R genotype responded poorly when treated with chemotherapy only and experienced the most benefit of the addition of cetuximab in terms of response rate. In line with this, Correale et al. demonstrated that activating *KRAS* mutations in colon cancer cell lines may correlate with a higher susceptibility to cetuximab-mediated ADCC [35]. Another study by Schlaeth et al. found that *KRAS* mutated tumor cells could be effectively killed by ADCC, indicating that mutated *KRAS* is not enough to confer resistance to antibody-mediated cell killing [36].

The conflicting findings in the different studies demonstrate the importance of sample size when studying the effect of polymorphisms in relation to clinical outcome. Moreover, the heterogeneity among the different studies, such as study design, ethnicity, previous and concomitant treatment, and the distribution of genotypes may also partly explain the discordance. Furthermore, the retrospective nature of most of the studies and the use of different endpoints may also contribute to the conflicting results. Additionally, Clynes et al. found the IgG1 antibodies trastuzumab and rituximab to engage in both activatory (*FCGR3A*) and inhibitory receptors (*FCGR2B*) and the *in vivo* activity of the antibodies may be more predictable by the ratio of *FCGR3A* to *FCGR2B* (A/I ratio) [37] which has not been investigated in the reported studies. Furthermore, all the studies have only tested two polymorphisms in only two genes involved in the ADCC mechanism. Also, other effector mechanisms of cetuximab may play a more important role, such as complement-dependent cytotoxicity, apoptosis and phagocytosis.

More importantly, ADCC may not play a correspondingly important role in metastatic cancer patients as demonstrated in *in vitro* models. ADCC has been shown to be markedly impaired with natural killer cell dysfunction in cancer patients with metastatic disease [38]. Moreover, the immune function in cancer patients may be impaired by the myeloablative effects of chemotherapy which may impair ADCC [39].

Primary tumors in the NORDIC VII study were screened for *KRAS* exon 2 (codons 12 and 13) mutations. Recent studies have though demonstrated that the selection of patients for anti-EGFR therapy may improve by considering *RAS* mutations other than *KRAS* exon 2 mutations (*NRAS* exons 2, 3, and 4 and *KRAS* exons 3 and 4) [16-18]. It is expected to find up to 17% mutations in the *KRAS* exon 2 wild-type population in the NORDIC VII cohort. We do not expect that the contribution of the additional mutations will considerably alter the outcome of the FCGR polymorphisms. Lack of this data is however a limitation of the present study.

## Conclusions

Patients with *KRAS* mutated tumors and the *FCGR2A* R/R genotype responded poorly when treated with chemotherapy only and experienced the most benefit of the addition of cetuximab in terms of response rate. The response rate for the *FCGR2A* R/R genotype was however not significantly larger than in the other two *FCGR2A* genotypes (H/R and H/H) in patients treated with Nordic FLOX and cetuximab. Moreover, there was no significant association between any of the *FCGR2A* genotypes and PFS or OS and the implication of this finding thus remains of uncertain clinical relevance. Many potential associations have been studied, and due to multiplicity a small number of low p-values would be expected to occur by chance even if no true associations exist. Furthermore, we found no significant association between any of the *FCGR3A* genotypes and response, PFS, or OS. Although our study has a larger sample size than most previously published studies, the sample size in the FCGR subgroups is still too low to obtain sufficient power and larger statistically powered studies to evaluate the significance of the FCGR polymorphisms are needed. Furthermore, the NORDIC VII cohort has limitations for studies of biomarkers predictive of cetuximab effect, as cetuximab did not add significant benefit to the Nordic FLOX regimen. In conclusion, we consider the *FCGR2A* and *FCGR3A* polymorphisms not to be currently useful predictive markers of cetuximab efficacy in mCRC.

## Competing interests

The authors declare that they have no competing interests.

## Authors’ contributions

AMD and JBK performed the genotyping. JBK analyzed the data and prepared the first draft of the manuscript. ES was involved in the interpretation of the data and contributed with statistical advice. KMT, TG, TI, CK, MKY, TF were responsible for recruitment of patients, blood sampling and clinical data collection. EHK was responsible for the biobanking. EHK brought the idea and organized the study. All authors read and approved the final manuscript.

## Pre-publication history

The pre-publication history for this paper can be accessed here:

http://www.biomedcentral.com/1471-2407/14/340/prepub
